# Supplementary material for: Prediction of cycloplegic refraction for noninvasive screening of children for refractive error
Source: PLoS One. 2021 Mar 15;16(3):e0248494. doi: 10.1371/journal.pone.0248494 (PMC7959391; doi:10.1371/journal.pone.0248494)
Supplement: Attachment — Submitted filename: Response to Reviewers.docx [file pone.0248494.s001.docx]

**Response to Reviewers**

We thank the reviewers for their helpful comments on our manuscript, our specific responses to which are as follows.

**Response to Reviewer #1**

*A welll performed study.
Do you think your prediction models would vary in different ethnic groups considering the high prevalence of myopia in Asia and hyperopia elsewhere?*

**Response:** The entry criteria we applied to the study did not include refraction of the candidate subjects. Ocular biometry with IOLMaster provides axial length of the eyeball and thus allows identification of individuals with extreme values of this parameter. Indeed, subjects with ocular refraction between –11.0 D and +10.0 D were enrolled. Given that we did not exclude individuals with high myopia or high hyperopia, we believe that our prediction formulas will be applicable to all ethnic groups, even those that tend to have a particular deviation of refraction. However, we did not test our prediction formulas specifically in subjects with extremely deviated refraction such as excessive myopia. The accuracy of the formulas for individuals with extremely deviated refraction will thus need to be validated in a future study.

*Can these formulas be incorporated or programmed in ocular biometry machines ?*

**Response:** We plan to talk to companies that produce ocular biometry instruments about the possibility of equipping them with our refraction prediction system, especially for the determination of refractive error in children.

*Line Numbering would have helped greatly with the review.*

**Response:** We apologize for the lack of line numbering, which we have now included in the revised manuscript.

*Grammatical errors:
Abstract:
line 7: "as well as.." replace by"* *in addition several noncycloplegic parameters were assessed using ocular biometry."*

**Response:** We made the suggested change. (lines 8 and 9)

*line 12: " were eye" instead of "was"*

**Response:** We could not find this in the original text.

*line 16: "and" instead of 'or"*

**Response:** Done. (line 13)

*Introduction:
page 5 line 13: rephrase "precise measurement"*

**Response:** We replaced with “assessment.” (line 56)

*page 6 line 6: "than was measured" replace by " than noncycloplegic..."*

**Response:** We replaced with “than was noncycloplegic.” (lines 67 and 68)

*Collection of clinical information:
page 8 line 12: remove "the" correct to "under noncycloplegic condition..."*

**Response:** We replaced with “under noncycloplegic conditions.” (line 103)

*Discussion
page 16: line 15: "of note" replace by "* *it is worthy to note that "*

**Response:** Done. (line 236)

*page 17: line 1: "In particular" needs rephrasing*

**Response:** We removed “In particular.” (line 240)

*line 11: remove " by our models"*

**Response:** Done. (line 250)

*page 18: line 11: "than is that" change to " than that"*

**Response:** Done. (line 267)

*line 15: remove word "thus"*

**Response:** Done. (line 273)

*line 16 &17: waht do you mean by "ocular stimulation"?*

**Response:** We replaced with “ocular irritation.” (lines 274 and 275)

*Page 19: line 14: " however" should be placed at beginning of sentence.*

**Response:** Done. (line 288)

*Line 14: Rephrasing needed maybe like "* *there are some challenges while performing biometery in children"*

**Response:** We replaced with “there are some challenges to performing ocular biometry in children.” (line 289)

*page 20: line 1: remove word "here".*

**Response:** Done. (line 294)

*line 3: provide instead of " provided"*

**Response:** Done. (line 296)

*line 4: rephrasing needed maybe to " under cycloplegic condition while avoiding..."*

**Response:** Changed to “under the cycloplegic condition while avoiding.” (line 297)

*line 5: "or with the failure to ....." start new sentence and rephrase.*

**Response:** Done. (Line 298)

**Response to Reviewer #2**

*This study tried to introduce a prediction formula for cycloplegic refraction in children, based on non-cycloplegic ocular biometry parameters. This can avoid the difficulties and the potential hazards encountered with performing cycloplegic refraction, allowing for easier screening and management of amblyopia. The following comments are proposed to improve the work.
Major comments:
1. Abstract: Please mention the study design in the “abstract”.*

**Response:** As suggested, we have now mentioned the retrospective study design in the abstract (line 3).
 *2. Methods:
• it would be more informative for the reader if you begin the “Methods” section by describing the study design.*

**Response:** We have now begun the Subjects and Methods section by stating the retrospective design of the study (line 77).

*• The study design is not clear: Was it totally retrospective? was all participants’ data retrieved by chart review? So, for what purpose did children undergoing refractive error evaluation have routine ocular biometry?*

**Response:** The study was totally retrospective. As shown in the subject flow diagram in Figure 1, we reviewed the clinical data for 3321 children and enrolled 617 of them as subjects based on the entry and exclusion criteria. Most of the excluded children lacked clinical data, usually that for ocular biometry. Given that we have been focused on the efficacy of ocular biometry for determination of axial length, which is closely related to the refractive error in children, we often performed ocular biometry examinations in children.

*• Page 13: regarding these 2 sentences “Statistical analysis revealed that the P value for the multiple regression equation for cycloplegic spherical refraction and predicted spherical refraction was <0.001.” and “Statistical analysis revealed that the P value for the multiple regression equation for cycloplegic cylinder refraction and predicted cylinder refraction was <0.001”…….What statistical test or parameter did you use for comparison between predicted and measured refraction?*

**Response:** We applied Pearson’s correlation coefficient for these comparisons, as is described in the “Statistical analysis” subsection of Subjects and Methods.

*• Regarding comparison group: This group has the same inclusion and exclusion criteria of the validation group. What did this third group add? Why could not such comparison be performed on the validation group?*

**Response:** As indicated by the reviewer, we could have performed the comparison analysis for the subjects in the validation and comparison groups combined. However, we planned on performing the study in three steps: (1) establishment of the prediction formulas, (2) validation of the prediction formulas, and (3) comparisons between predicted values and cycloplegic or noncycloplegic measurements. We selected the subjects for each analysis on the basis of the date on which they visited the hospital. The application of these steps revealed a consistent relation among cycloplegic, noncycloplegic, and predicted values. However, given the reviewer’s comment, we omitted Figures 4 to 7 of the original manuscript (showing correlations between cycloplegic and either predicted or noncycloplegic values in the comparison group) in the revised version, and instead just show the data for these comparisons in Tables 5 and 6.

*3. Results: regarding figures: please add a brief description of the figure to figure legends; to allow readers to understand the key message of the figure without referring to the text.*

**Response:** As suggested, we have now added short descriptions to the figure legends.

*Minor comments:
1. Page 18: “Children receiving these eyedrops may also experience ocular stimulation, which may result in a lack of patient cooperation.” What does “ocular stimulation” refer to?*

**Response:** We changed “ocular stimulation” to “ocular irritation” (line 273).

*2. Page 19: “In the present study, we performed ocular biometry to obtain precise values for axial length, anterior chamber depth, lens thickness, corneal refractive power, and corneal astigmatism within 10 s for each eye.” …. “seconds” is better to be written in full.*

**Response:** We changed “s” to “seconds” (lines 281 and 290).
